# The improvement of photocatalysis O_2_ production over BiVO_4_ with amorphous FeOOH shell modification

**DOI:** 10.1038/s41598-019-54940-2

**Published:** 2019-12-13

**Authors:** Ying Zhang, Lei Shi, Zhongxing Geng, Tieqiang Ren, Zhanxu Yang

**Affiliations:** College of Chemistry, Chemical Engineering and Environmental Engineering, Liaoning Shihua University, Fushun, 113001 China

**Keywords:** Energy science and technology, Materials science, Nanoscience and technology

## Abstract

A novel amorphous FeOOH modified BiVO_4_ photocatalyst (A-FeOOH/BiVO_4_) was successfully produced and characterized by various techniques. The results showed that amorphous FeOOH with about 2 nm thickness evenly covered on BiVO_4_ surface, which caused resultant A-FeOOH/BiVO_4_ exhibiting higher visible light photocatalytic performance for producing O_2_ from water than BiVO_4_. When the covered amount of amorphous FeOOH was 8%, the resultant photocatalyst possessed the best photocatalytic performance. To find the reasons for the improvement of photocatalytic property, electrochemical experiments, DRS, PL and BET, were also measured, the experimental results indicated that interface effect between amorphous FeOOH and BiVO_4_ could conduce to migration of photogenerated charge, and exhibit stronger light responded capacity. These positive factors promoted A-FeOOH/BiVO_4_ presenting improved the photocatalytic performance. In a word, the combination of amorphous FeOOH with BiVO_4_ is an effective strategy to conquer important challenges in photocatalysis field.

## Introduction

Semiconductor photocatalysis has achieved keen attention in utilizing solar power to solve environments deterioration and energy crisis^1–3^. Hence, numerous photocatalyst materials were developed, including TiO_2_^4–6^, ZnO^7^, WO_3_^8–10^, CdS^11,12^, SnS_2_^13^, Ag based photocatalysts^14–16^, etc. However, the development of photocatalysts with high efficient is still a huge and continuous undertaking.

The Bi-based photocatalyst materials, such as Bi_2_O_3_^17^, BiOCl^18^, BiOBr^19^, BiOI^20^, BiVO_4_^21–23^, Bi_2_WO_6_^24^, and Bi_2_MoO_6_^25^, have obtained great attention. Among these as-prepared photocatalysts, BiVO_4_ with strong visible light response capacity and good stability has been extensively investigated in environmental remediation and water splitting^26–31^. Nevertheless, a big problem that affects the photocatalytic property of BiVO_4_ is its unsatisfied charge carrier separated efficiency. To conquer this problem, many researchers had developed some methods for modifying BiVO_4_. Cao *et al*. successfully prepared Au-BiVO_4_ photocatalyst, which could present much higher visible-light photocatalytic performance for wastewater treatment and clean energy product than the individual BiVO_4_^32^. Except Au modification, Pd, AuPd and CoPd were used to modify BiVO_4_ to enhance its photocatalytic property^33–35^. However, these noble metals or noble metal alloys were high cost so that this method was difficult to be wide application. Hence, it was necessary to develop an economical and convenient method to modify BiVO_4_.

Recently, amorphous semiconductor materials have been exploited and exhibited specific photocatalytic property^36,37^. Compared to crystals, amorphous semiconductor materials exist a most remarkable advantage that it possesses much smaller band gap than their crystalline counterparts, which conduces them to present more expansive light absorption range, which is an important condition for conversing solar energy^37^. However, amorphous semiconductor materials exhibited the short-range atomic order, and existed a number of defects, which could become the charge recombination centers, causing themselves inactive or weak performance. So could numerous defects existed on amorphous semiconductor materials as the electrons capturer be applied for modifying other photocatalysts? This view is very significative and interesting. Hence, considering the wide light responded property and cost, Fe based semiconductors enter into our view. Among Fe based semiconductors, FeOOH exhibits extensive visible light response capacity, which caused FeOOH coupled with other photocatalysts to modify the visible-light-irradiation photocatalytic property, some FeOOH with certain crystalline phase was used to modify photocatalyst materials, such as β-FeOOH/TiO_2_^38^, β-FeOOH/g-C_3_N_4_^39^, α-FeOOH/AgVO_3_^40^, etc. Actually, FeOOH contained various crystalline phases, including α-phase, β-phase, δ-phase, γ-phase, and amorphous phase. Among these different crystalline phase FeOOH, amorphous FeOOH could exhibit excellent oxygen evolution rate by photoelectrochemical and superior pseudocapacitive performance^41,42^. However, it is relative lack of report about amorphous FeOOH as a modifier to be used in photocatalysis. Hence, combined with above proposed view, it is essential to investigate the role of amorphous FeOOH modifier.

Herein, a novel amorphous FeOOH modified BiVO_4_ was successfully prepared, and photocatalytic performance for producing O_2_ from water was investigated. It could be found that, after amorphous FeOOH evenly covered the surface of BiVO_4_, as-prepared photocatalysts exhibited better migration of photogenerated charges, and stronger visible light responded activity. These positive factors promoted A-FeOOH/BiVO_4_ presenting improved the photocatalytic performance. Hence, this work shows an effective and simple modified method for designing and preparing highly efficient photocatalysis materials.

## Experimental

### The synthesis of catalysts

To obtain BiVO_4_ material, in a beaker, Bi(NO_3_)_3_·5H_2_O (5 mmol) dissolved in HNO_3_ solution (5 mL 3 mol·L^−1^) and ethylene glycol (20 mL) mixed solution. Then in other beaker, NH_4_VO_3_ (5 mmol) and 0.25 g sodium dodecylbenzenesulfonate (SDBS) were dissolved in hot water (20 mL). After stirred for 30 min, above two solution mixed, and the pH of solution was adjusted to 5 using NaOH solution. Stirring for 60 min, obtained suspension solution was placed into high pressure reactor with PTFE liner, maintained at 180 °C for 24 h. After filtration, wash and desiccation, BiVO_4_ was prepared.

Amorphous FeOOH/BiVO_4_ was prepared as follows: BiVO_4_ (400 mg) was mixed into 40 mL deionized water containing FeCl_3_·6H_2_O and NH_4_HCO_3_ (The molar ratio of FeCl_3_·6H_2_O and NH_4_HCO_3_ is 1:3). Stirring for 60 min, the solid powder was obtained through centrifugation, wash and desiccation. According to the theoretical content of amorphous FeOOH in amorphous FeOOH/BiVO_4_, the obtained powder catalysts were marked as A-FeOOH/BiVO_4_(2 wt%), A-FeOOH/BiVO_4_(5 wt%), A-FeOOH/BiVO_4_(8 wt%) and A-FeOOH/BiVO_4_(10 wt%), respectively.

### Characterizations and photocatalytic experiment

Supporting Materials showed their details.

## Results and Discussion

**Figure 1:**
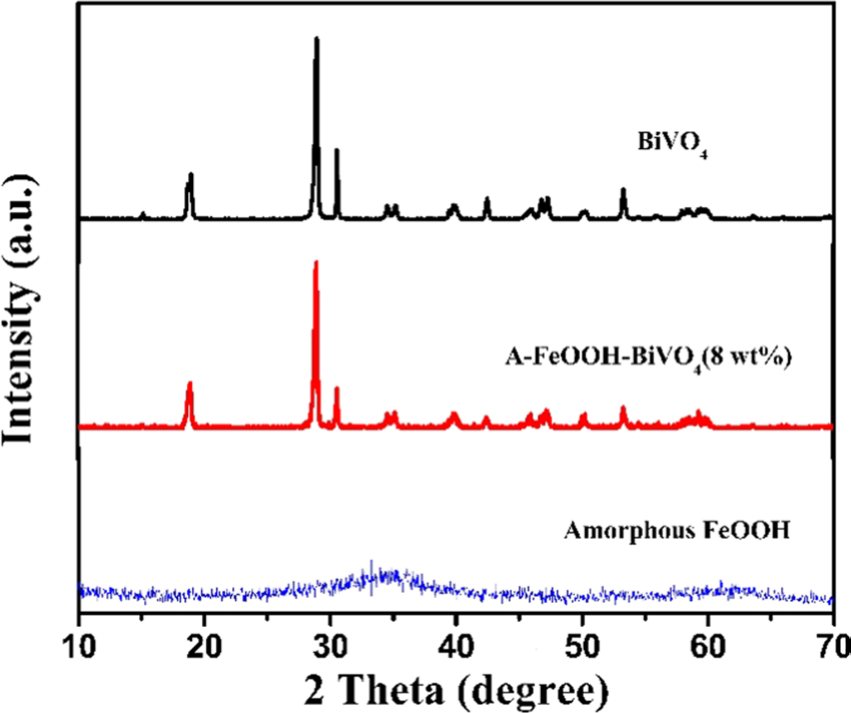
The X-ray diffraction patterns of as-prepared photocatalysts.

Figure 1 demonstrates the XRD of resultant photocatalysts. As can be observed, resultant BiVO_4_ exhibited highly crystalline, and there were some mainly diffraction peaks at 2θ of 18.6°, 28.9°, 30.5°, 34.4°, 35.3°, 39.4°, 42.3°, 46.1°, 46.6°, 47.3°, 53.3°, 58.3° and 59.9^o^, which indexed (101), (013), (112), (200), (020), (211), (105), (123), (204), (024), (301), (303) and (224) diffraction planes of monoclinic BiVO_4_^43^. For amorphous FeOOH, there was no obvious diffraction peak to be found. Hence, in A-FeOOH/BiVO_4_(8 wt%), XRD diffraction peaks of BiVO_4_ could only be detected. The XRD results exposed that amorphous FeOOH had little impact the crystal phase of BiVO_4_. Moreover, no other diffraction peak was found, meaning that resultant samples possessed the high purity.

**Figure 2:**
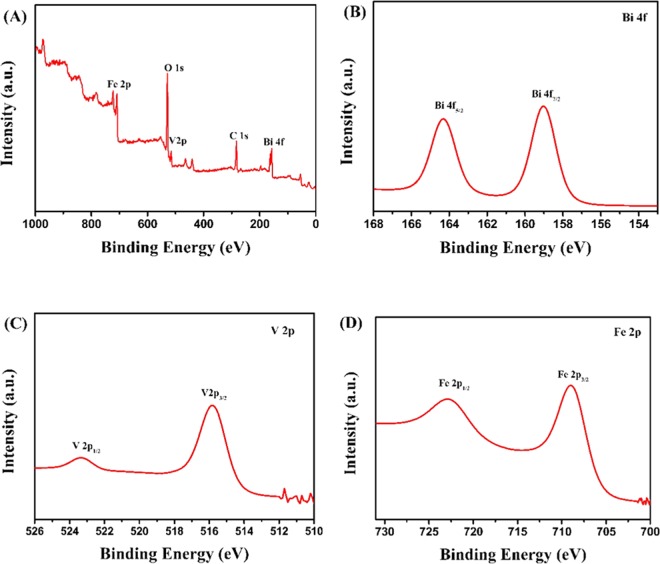
The XPS high resolution (**A**) survey spectra, (**B**) Bi 4f, (**C**) V 2p and (**D**) Fe 2p spectra of A-FeOOH/BiVO_4_(8 wt%).

Whereafter, the XPS of A-FeOOH/BiVO_4_(8 wt%) was further investigated. As revealed in Fig. 2A, Bi, V, O and Fe could be found in the survey XPS spectra, according with the composition of material. Bi 4 f XPS spectrum (Fig. 2B) presented 164.3 eV and 159.1 eV two peaks that attributed to Bi 4f_5/2_ and Bi 4f_7/2_ in BiVO_4_^44,45^. V 2p XPS spectrum (Fig. 2C) had two peaks at 516.3 eV and 524.3 eV, matching with V 2p_1/2_ and V 2p_3/2_ in BiVO_4_^46^. Figure 2D (Fe 2p XPS spectrum) shows 724.2 (Fe 2p_1/2_) and 710.8 eV (Fe 2p_3/2_) that were consistent with FeOOH^47^. Hence, the results of XPS further confirmed that sample contained FeOOH and BiVO_4_, consistent to the XRD.

**Figure 3:**
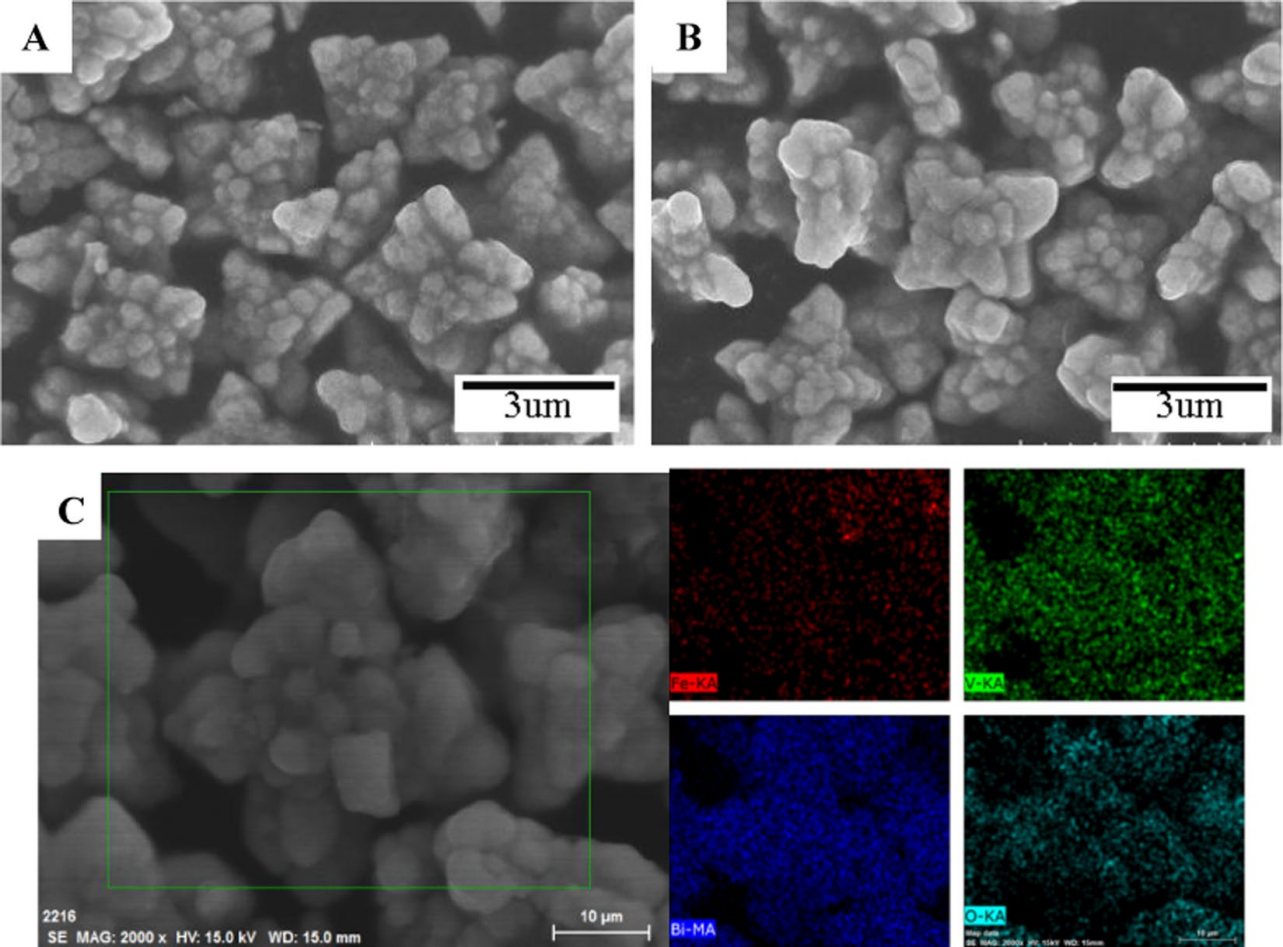
The SEM images of (**A**) BiVO_4_ and (**B**) A-FeOOH/BiVO_4_(8 wt%) and (**C**) SEM-EDS mapping images of A-FeOOH/BiVO_4_(8 wt%).

The morphologies of BiVO_4_ and A-FeOOH/BiVO_4_(8 wt%) were seen through SEM. As revealed in Fig. 3, BiVO_4_ and A-FeOOH/BiVO_4_(8 wt%) exhibited similar star-shaped particles with the size of about 3.5 μm, which demonstrated that introduced amorphous FeOOH did not influence on the feature of BiVO_4_. In addition, the elemental compositions of the resultant A-FeOOH/BiVO_4_(8 wt%) were measured through EDS mapping analysis. The related element mapping images were exhibited in Fig. 3C. Clearly, Fe, V, Bi and O only appeared in observed view, meaning the successful preparation of A-FeOOH/BiVO_4_.

**Figure 4:**
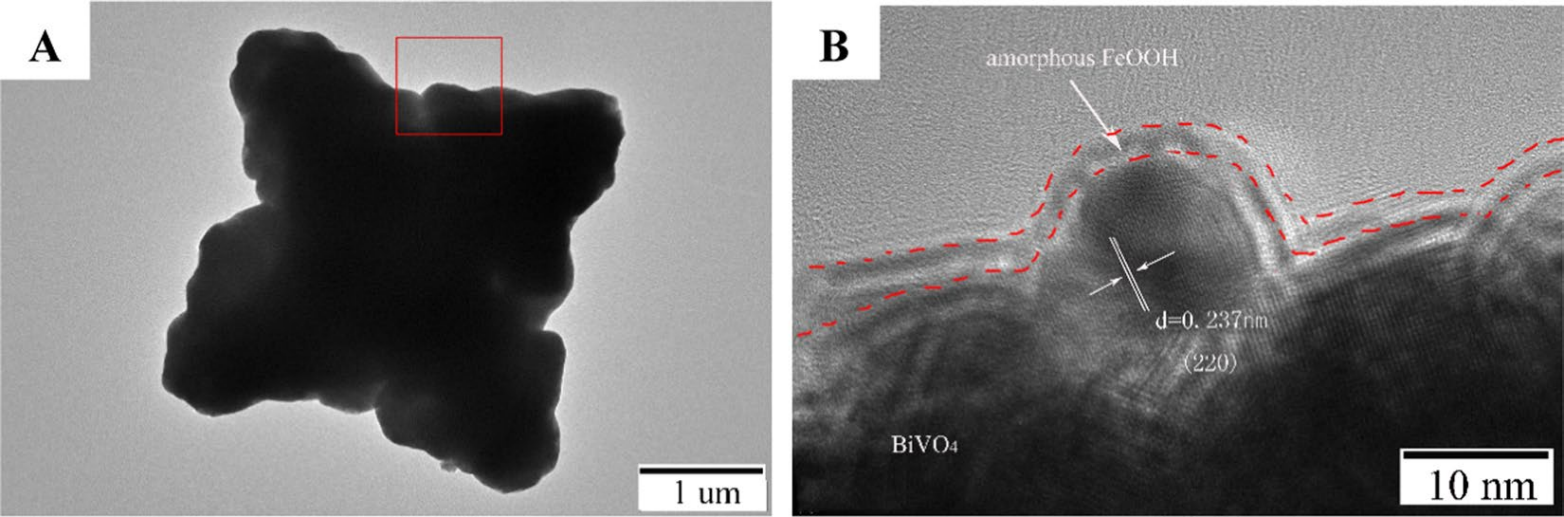
The (**A**) TEM and (**B**) HRTEM images of A-FeOOH/BiVO_4_(8 wt%).

Subsequently, to further observe the microstructure of A-FeOOH/BiVO_4_, transmission electron microscopy were also analyzed. As revealed in Fig. 4A, A-FeOOH/BiVO_4_(8 wt%) exhibited the similar star-shaped particles, which was consistent with the SEM. Clearly, amorphous FeOOH could not be observed at low resolution TEM image. As a result, the HRTEM of A-FeOOH/BiVO_4_ (Fig. 4B) was provided. The lattice fringe spacing of 0.237 nm attributed to BiVO_4_ (220) was obviously observed. Moreover, the thickness of ultrathin amorphous FeOOH nanolayers was about 2 nm, and evenly adhered on the surface of BiVO_4_. Amorphous FeOOH nanolayers did not reveal a lattice spacing, demonstrating traditional amorphous structure.

**Figure 5:**
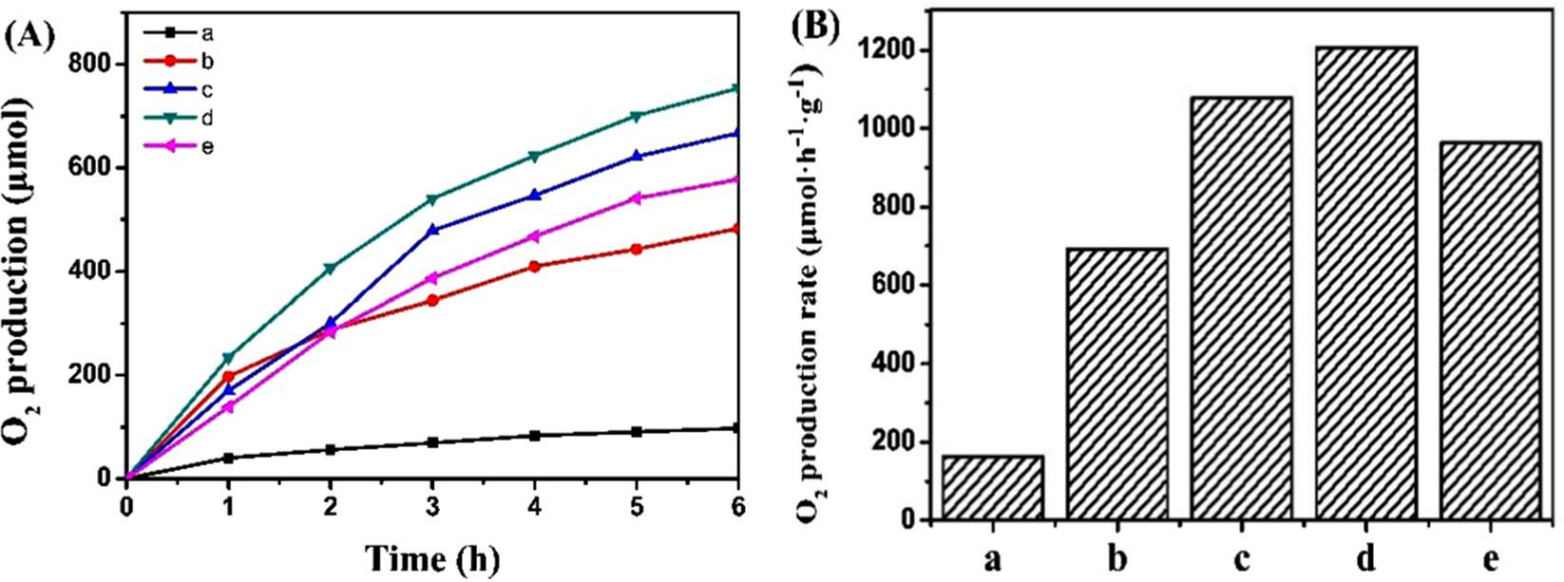
(**A**) Photocatalytic evolution O_2_ curves and (**B**) OER over various samples, (a) BiVO_4_, (b) A-FeOOH/BiVO_4_(2 wt%), (c) A-FeOOH/BiVO_4_(5 wt%), (d) A-FeOOH/BiVO_4_(8 wt%) and (e) A-FeOOH/BiVO_4_(10 wt%).

The photocatalytic property over resultant photocatalysts was measured through producing O_2_ from slitting water. Before producing O_2_, to improve oxygen evolution rate, the sacrificial agent need be added. The previous report indicated that BiVO_4_ could exhibit better photocatalytic performance for producing O_2_ in the NaIO_4_ solution than that in the AgNO_3_ solution^48^. Meanwhile, to verify this result, we also measured the photocatalytic performance for producing O_2_ over A-FeOOH/BiVO_4_ photocatalyst in the same concentration NaIO_4_ or AgNO_3_ solution. As shown in Fig. S1, A-FeOOH/BiVO_4_ photocatalyst in the NaIO_4_ solution could present high oxygen evolution rate (OER) than A-FeOOH/BiVO_4_ photocatalyst in the AgNO_3_ solution. Hence, in this system, we selected NaIO_4_ solution as the sacrificial agent. Then the results of oxygen evolution over all of photocatalysts were shown in Fig. 5A. For a series of resultant A-FeOOH/BiVO_4_ photocatalysts, O_2_ could be persistently produced with reaction time prolonging. Compared with pure BiVO_4_, resultant A-FeOOH/BiVO_4_ photocatalysts could clearly display the improvement of photocatalytic capacity for producing O_2_. When loading amount of amorphous FeOOH was 8%, the sample had the optimal catalytic rate of O_2_ evolution. Then Fig. 5B gives their OER. Their OER were 162.3, 691.7, 1077.1, 1206.3 and 962.8 μmol h^−1^ g^−1^ for pure BiVO_4_, A-FeOOH/BiVO_4_(2 wt%), A-FeOOH/BiVO_4_(5 wt%), A-FeOOH/BiVO_4_(8 wt%) and A-FeOOH/BiVO_4_(10 wt%), respectively. The OER of A-FeOOH/BiVO_4_(8 wt%) was around 7.4 folds more than pure BiVO_4_. In addition, the O_2_ production rates of different materials in previous reports have been in Table 1^49–52^, and compared with this work. Obviously, as-prepared A-FeOOH/BiVO_4_(8 wt%) in this work could present excellent advance.

**Table 1:** Comparison of photocatalytic activity in previous reports and this work.

Samples	Light source	Application	Catalyst mass	Performance	References
WO_3_	Visible light	O_2_ production	200 mg	246.2 μmol in 5 h	^49^
BiVO_4_ nano-leaves	Visible light	O_2_ production	100 mg	300 μmol in 5 h	^50^
BiVO_4_	Visible light	O_2_ production	1000 mg	186 µmol in 6 h	^51^
a-Fe_2_O_3_	Visible light	O_2_ production	10 mg	10.5 µmol in 15 h	^52^
A-FeOOH/BiVO_4_(8 wt%)	Visible light	O_2_ production	100 mg	723 µmol in 6 h	This work

**Figure 6:**
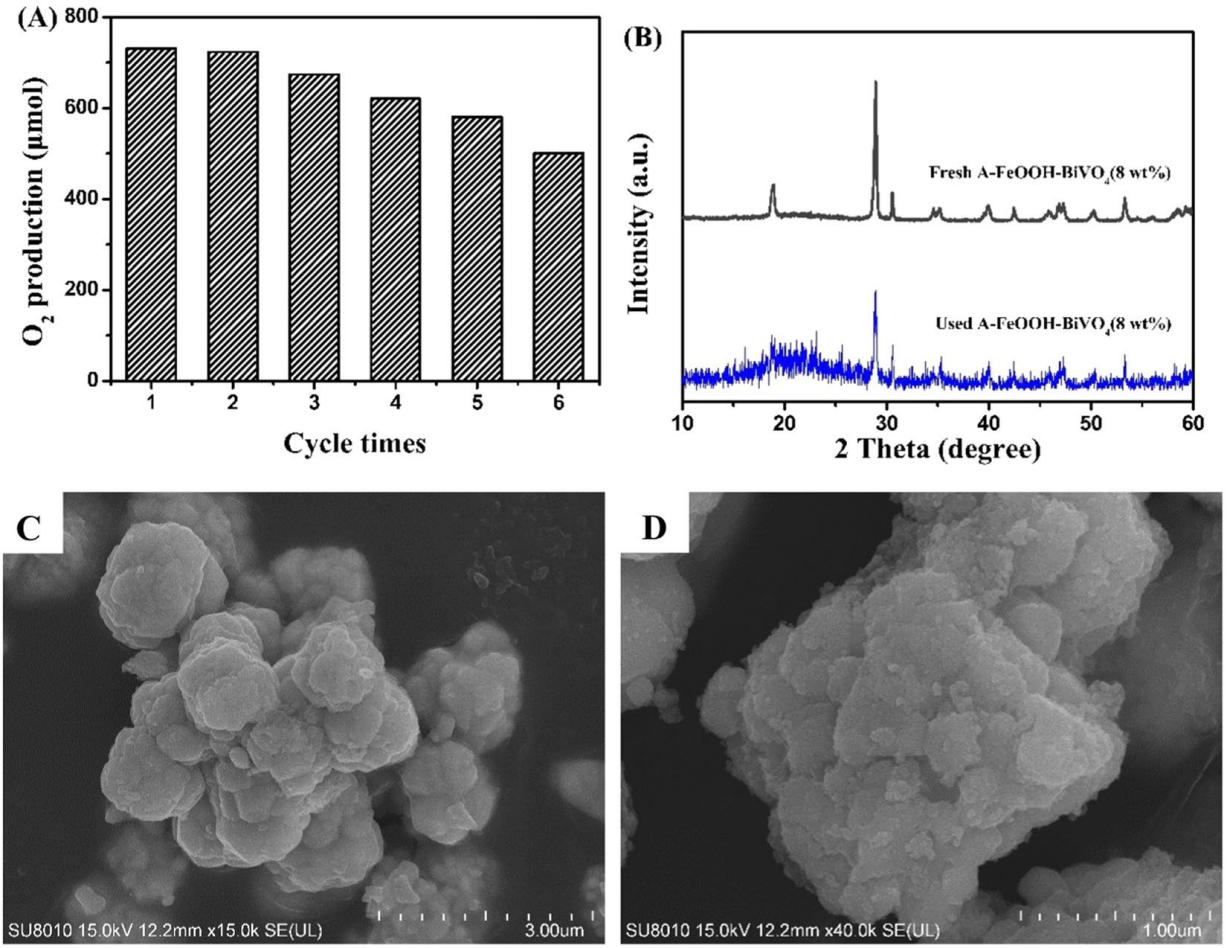
(**A**) Stability test in O_2_ photosynthesis for A-FeOOH/BiVO_4_(8 wt%), (**B**) the XRD of fresh A-FeOOH/BiVO_4_(8 wt%) and used A-FeOOH/BiVO_4_(8 wt%) and (**C,D**) The SEM images of used A-FeOOH/BiVO_4_(8 wt%).

Furthermore, as we known, the stability is a very significant index to appraise its ability. Thus, recycling tests for producing O_2_ over A-FeOOH/BiVO_4_(8 wt%) were investigated. As demonstrated in Fig. 6A, the A-FeOOH/BiVO_4_(8 wt%) presented relative stable OER, after 6 times cycled experiments, as-prepared A-FeOOH/BiVO_4_(8 wt%) still presented 70% photocatalytic activity of fresh sample. Besides, the XRD of used A-FeOOH/BiVO_4_(8 wt%) was measured in Fig. 6B. Its XRD did not have obviously change in comparison to fresh A-FeOOH/BiVO_4_(8 wt%). And the morphology of used sample was also observed in Fig. 6C,D. It can be observed that used sample exhibited a certain aggregate in comparison to fresh sample, which might result in the declined photocatalytic property. Besides, the whole morphology did not present great change.

Obviously, it was found that covered amorphous FeOOH could effectively modify BiVO_4_ to improve catalytic activity. Why? To find the reason for promoting effect, some analyzed instruments were tested to investigated light response capacity, the photoinduced charge separated rate and surface area of as-prepared photocatalysts, which are deemed as the main factors to effect on the photocatalytic performance^53–55^.

**Figure 7:**
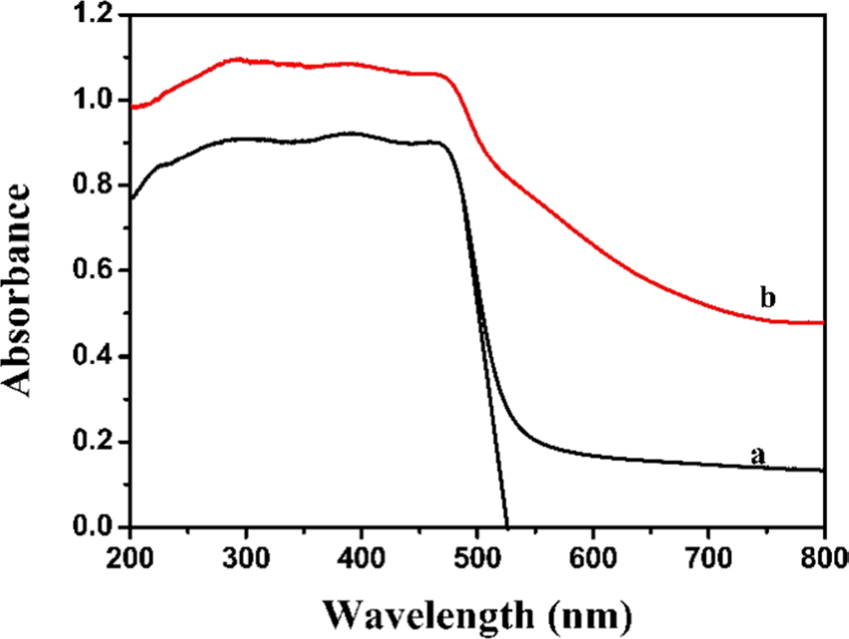
The UV-Vis absorption spectrum of (**a**) BiVO_4_ and (**b**) A-FeOOH/BiVO_4_ (8%).

Figure 7 presents the UV-Vis absorption spectrum of resulted BiVO_4_ and A-FeOOH/BiVO_4_(8 wt%). Pure BiVO_4_ presented remarkable light absorption between 200 and 800 nm. The absorption edge was 525 nm. Band gap was 2.36 eV. After covered by amorphous FeOOH, the light absorption capacity of as-obtained A-FeOOH/BiVO_4_(8 wt%) gained the obvious enhancement. Hence, the enhanced photocatalytic property might be anticipated.

**Figure 8:**
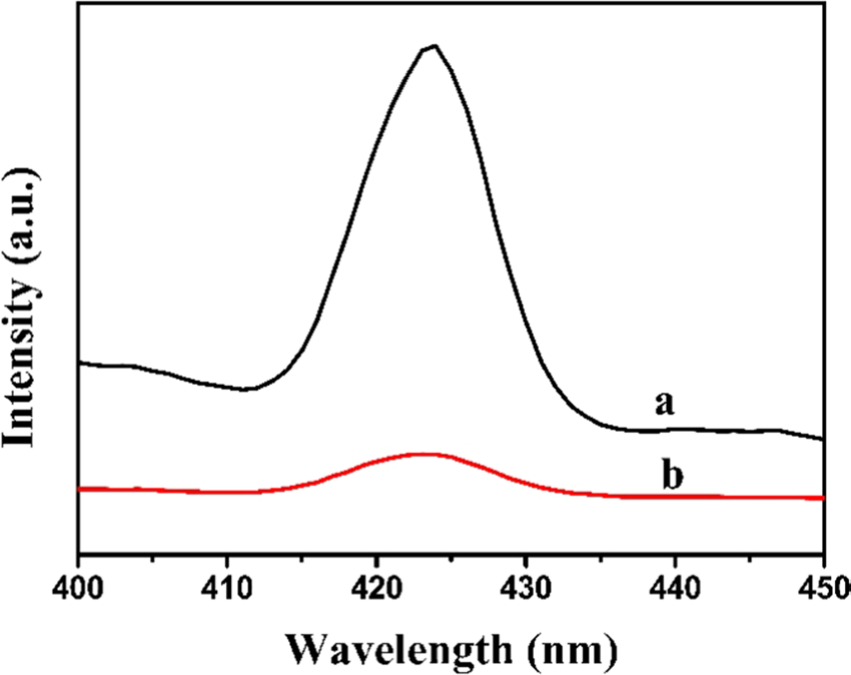
The photoluminescence spectra of (**a**) BiVO_4_ and (**b**) A-FeOOH/BiVO_4_ (8%).

Photoluminescence (PL) property could analyze the separation and transfer efficiency of photoinduced charges^56^. Therefore, the PL of BiVO_4_ and A-FeOOH/BiVO_4_ (8%) were measured in Fig. 8. The emission band intensity of A-FeOOH/BiVO_4_ (8%) was clearly declined in comparison of pure BiVO_4_. This result implied that covered amorphous FeOOH could effectively reduce the recombined efficiency of photoinduced electrons and holes, conducing to improve photocatalytic property^57^.

**Figure 9:**
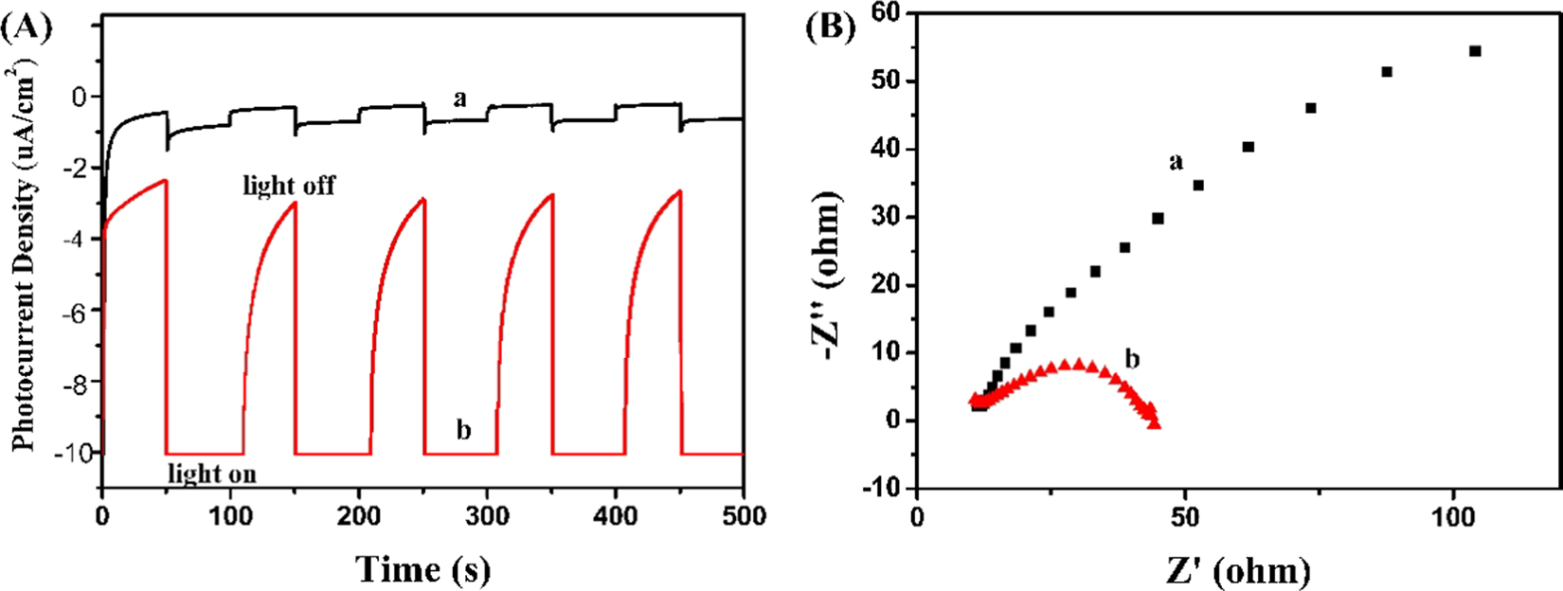
(**A**) Transient photocurrent property and (**B**) EIS of (a) BiVO_4_ and (b) A-FeOOH/BiVO_4_ (8%).

Subsequently, electrochemistry measurements (photocurrent and EIS measurement) were used to assess the separated and transfer efficiency of photoinduced charges. Currently, the stronger photocurrent manifests the more effective separation and transfer rate of photo-charges^58,59^. As illustrated in Fig. 9A, pure BiVO_4_ and A-FeOOH/BiVO_4_(8 wt%) could produce certain intensity photocurrent signals. And the order of photocurrent intensity from strong to weak was A-FeOOH/BiVO_4_(8 wt%) > pure BiVO_4_. Evidently, resultant A-FeOOH/BiVO_4_(8 wt%) exhibited the better photocurrent intensity, revealing that the separated rate of photogenerated charges for A-FeOOH/BiVO_4_(8 wt%) photocatalyst was significantly improved by covering amorphous FeOOH.

Then EIS techniques were further used to estimate charge separation property. Figure 9B gives the EIS of pure BiVO_4_ and A-FeOOH/BiVO_4_(8 wt%). Small frequency semicircle radius exposes a better charge transfer rate. As demonstrated in Fig. 9B, the semicircle radius of A-FeOOH/BiVO_4_(8 wt%) was shorter than that of pure BiVO_4_, meaning that A-FeOOH/BiVO_4_(8 wt%) possessed a higher separation and transport rate of photogenerated charges.

**Figure 10:**
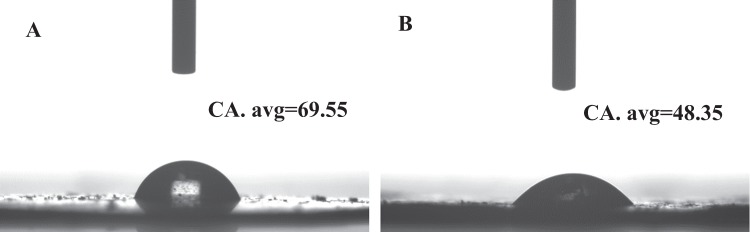
Water contact angle of (**A**) BiVO_4_ and (**B**) A-FeOOH/BiVO_4_(8 wt%).

Besides, the BET surface area of pure BiVO_4_ and A-FeOOH/BiVO_4_(8 wt%) were 4.9 and 5.2 m^2 ^g^−1^, respectively. The addition of amorphous FeOOH had little effect on the surface area of photocatalyst. Additionally, the surface hydrophilic property of photocatalyst was performed to measure interact with the water. As presented in Fig. 10A,B, water contact angle (CA) of BiVO_4_ (69.55°) and A-FeOOH/BiVO_4_(8 wt%) (48.35°) was measured. This result meant that the covered amorphous FeOOH made BiVO_4_ possess water favorable wetting capacity, providing a good chance to oxidate H_2_O in aqueous environment^60^.

Hence, combined with above analyzed results, it was found that, the main reason that as-prepare A-FeOOH/BiVO_4_ possessed the better photocatalytic performance than BiVO_4_ could be explained that the former exhibited higher efficiency for separation of photogenerated charges, and stronger strong visible responded activity compared with the latter. Hence, the remarkable improvement of photocatalytic capacity for producing O_2_ was obtained.

Whereafter, to speculate the photocatalytic mechanism, the energy structure of BiVO_4_ might be calculated^61^

1 $${\rm{Eg}}={{\rm{E}}}_{{\rm{VB}}}-{{\rm{E}}}_{{\rm{CB}}}$$

2 $${{\rm{E}}}_{{\rm{VB}}}={\rm{X}}-{{\rm{E}}}_{{\rm{e}}}+1/2{\rm{Eg}}$$

Herein Eg, E_VB_, E_CB_, X, E_e_ are band gap of photocatalyst, the valence band potential, conduction band potential, electro negativity of component atoms, hydrogen scale (4.5 eV), respectively. Here, for BiVO_4_, X is 6.15 eV^62^, Eg is 2.36 eV (Fig. 7). Hence, the CB and VB potentials of BiVO_4_ were respective 0.47 and 2.83 eV.

**Figure 11:**
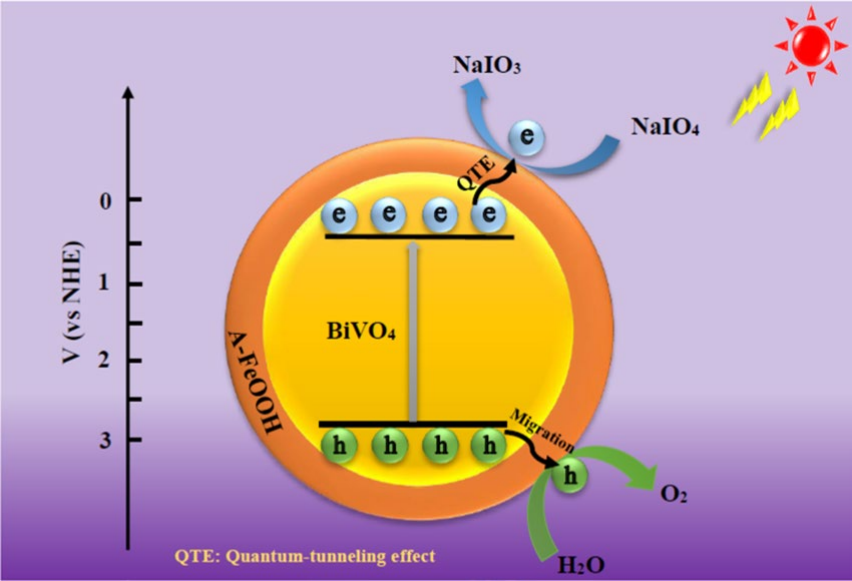
The proposed mechanism of photocatalytic water oxidation over A-FeOOH/BiVO_4_ photocatalyst.

Finally, in Fig. 11, a probable photocatalytic mechanism for A-FeOOH/BiVO_4_ was presented. BiVO_4_ could generate electrons and holes under light irradiation. Due to quantum-tunneling effect (QTE)^63,64^, formed charges from the conduction band of BiVO_4_ could transfer through amorphous FeOOH, or cationic vacancy network in amorphous FeOOH phase^65^. Then NaIO_4_ as the electrons sacrificial agent consumed electrons. Because the cationic vacancy might be also activated by trapping hole^66^, then holes remained on the VB of BiVO_4_ would migrate to amorphous FeOOH surface to produce O_2_. Hence, in this process, the existence of amorphous FeOOH could boost the separation of photoinduced charges for A-FeOOH/BiVO_4_ system, obtaining the enhancement of photocatalytic performance.

## Conclusions

We successfully produced a novel amorphous FeOOH modified BiVO_4_, and investigated it photocatalytic performance for producing O_2_ from water. It could be found that, amorphous FeOOH modified BiVO_4_ exhibited higher migration rate of photogenerated charges, and strong visible responded capacity than BiVO_4_, which resulted that amorphous FeOOH modified BiVO_4_ could present better photocatalytic property than BiVO_4_, and kept excellent performance and structure stability. Hence, this work provides a simple and inexpensive modified method for design and synthesis of effective photocatalysts.

## Supplementary information

Supporting information
