# Supplementary material for: The improvement of photocatalysis O2 production over BiVO4 with amorphous FeOOH shell modification
Source: Sci Rep. 2019 Dec 13;9:19090. doi: 10.1038/s41598-019-54940-2 (PMC6911067; doi:10.1038/s41598-019-54940-2)
Supplement: Supplementary file 1 — Supporting information [file 41598_2019_54940_MOESM1_ESM.docx]

**Supporting Information**

**The improvement of photocatalysis O_2_ production over BiVO_4_ with amorphous FeOOH shell modification**

Ying Zhang ^a^, Lei Shi ^a^*, Zhongxing Geng ^a^, Tieqiang Ren ^a^, Zhanxu Yang ^a^*

a: College of Chemistry, Chemical Engineering and Environmental Engineering, Liaoning Shihua University, Fushun 113001, China

*Corresponding authors: Lei Shi, shilei_hit@qq.com; Zhanxu Yang, yangzhanxu@lnpu.edu.cn

Tel: +86-24-56860958

**Material characterizations**

An X-ray powder diffractometer (Bruker D8 Advance) was used for detecting phase identification. The X-ray photoelectron spectroscopy (XPS) was measured on Thermo Fisher Scientific Escalab 250. The morphologies of photocatalysts were observed by scanning electron microscopy (SEM, Hitachi SU8010, Japan) with Energy dispersive spectrometry (EDS) elemental mapping analysis. The microtopographies were recorded by transmission electron microscopy (TEM, JEM-2010, Japan). The UV-vis diffuse reflectance spectra (DRS) were measured using Agilent UV-vis spectrometer (Cary 5000, America). The photoluminescence spectra (PL) were recorded through an Agilent fluorescence spectrometer (Cary Eclipse, America). A Quantachrome Autosorb-IQ2-MP BET surface area analyzer was used to measure surface area. Photo-electrochemical analysis and electrochemical impedance spectroscopic (EIS) measurements were detected through a DELIXI electrochemical station. In whole system, the counter electrode was Pt electrode. The reference electrode was the saturated calomel electrode. The sample films coated on ITO glasses were the working electrode. 0.1 M Na_2_SO_4_ was used as the electrolyte. The water contact angle (WCA) was determined by an optical contact angle and interface tension meter (KINO SL 200KB).

**Photocatalytic test**

The photocatalytic evolution O_2_ experiments over resultant photocatalysts were investigated in a Pyrex top-irradiation reaction vessel, which connected to a closed quartz glass gas-circulation system, 300W Xe lamp supplied light source. A 420 nm cutoff ﬁlter was used to cut off under 420 nm light. 100 mg powder catalyst was added in 100 mL NaIO_4_ solution (0.02 M). The reactant solution was evacuated by vacuum pump for 0.5 h to remove air before light illumination. In this system, the O_2_ was analyzed by gas chromatography, Ar was the carrier gas.


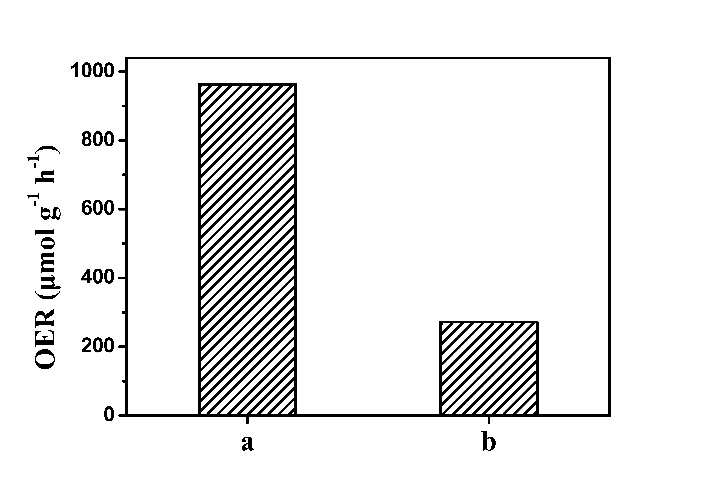


**Fig. S1** The OER of A-FeOOH/BiVO_4_(10 wt%) in (a) NaIO_4_ solution or (b) AgNO_3_ solution
